# Reporting Standards and Quality Assurance Methods for Pancreatoduodenectomy in Randomised Controlled Trials: A Structured Narrative Review

**DOI:** 10.3390/jcm15062455

**Published:** 2026-03-23

**Authors:** Abdullah K. Malik, Bishow B. Karki, Balaji Mahendran, John A. G. Moir, Shailesh V. Shrikhande, Andrew M. Smith, Deborah D. Stocken, Natalie S. Blencowe, Samir Pathak

**Affiliations:** 1 Department of HPB and Transplant Surgery, Freeman Hospital, Newcastle upon Tyne Hospitals NHS Foundation Trust, Newcastle upon Tyne NE7 7DN, UK; 2 Newcastle Fibrosis Research Group, Newcastle University, Newcastle upon Tyne NE1 7RU, UK; 3 Department of Pancreatobiliary Surgery, St James’s Hospital, Leeds Teaching Hospitals NHS Trust, Leeds LS9 7TF, UKsamir.pathak1@nhs.net (S.P.); 4 Department of HPB Surgery, Tata Memorial Hospital, Mumbai 400012, India; 5 Leeds Institute for Clinical Trials Research, University of Leeds, Leeds LS2 9JT, UK; 6 Centre for Surgical Research, Population Health Sciences, Bristol Medical School, Bristol BS8 2PN, UK; 7 Leeds Institute for Emergency General Surgery, St James’s Hospital, Leeds Teaching Hospitals NHS Trust, Leeds LS9 7TF, UK

**Keywords:** pancreaticoduodenectomy, randomised controlled trials, surgical quality assurance, operative standardisation

## Abstract

**Background:** Surgical interventions are complex and comprise multiple components, creating difficulties when considering how they might be described, standardised, and monitored (i.e., quality assurance) within randomised controlled trials (RCTs). Consolidated Standards of Reporting Trials – Non-Pharmacological Treatment (CONSORT-NPT) provides specific recommendations to improve the quality, transparency, and replicability of RCTs involving a surgical intervention. This structured narrative review explores and summarizes the reporting of quality assurance measures for surgical interventions in RCTs, using pancreatoduodenectomy (PD) as an exploratory case study. **Methods:** Searches for RCTs of PD were undertaken in PubMed, Medline, the Cochrane Central Register of Controlled Trials, and the Cochrane Database of Systematic Reviews from 2020 to 2024. Pancreatoduodenectomy (PD) was deconstructed into its constituent components (*n* = 40), and selected RCTs were scrutinised to explore reporting of quality assurance measures against the deconstructed components as described in CONSORT-NPT. **Results:** Of 189 screened articles, 37 RCTs were included, reporting on 5659 patients across 16 countries. No studies described all components of PD, and four did not report any components at all. Nine studies described some form of standardisation, and three measured adherence to standards, using intra-operative photographs. Minimum surgeon and centre volumes were specified in two and six trials, respectively. **Conclusions:** Quality assurance measures were poorly reported in selected RCTs involving PD, creating uncertainty in interpreting results. To enhance the design of future RCTs, a wider consensus regarding the core components of a PD is required. This will facilitate subsequent consideration of how these might need to be reported in future pancreatic surgical RCTs.

## 1. Introduction

Pancreatic cancer has poor long-term outcomes, with an overall five-year survival rate of approximately 10% [1]. Tumours are most commonly located in the head of the pancreas, and surgery—pancreaticoduodenectomy (PD)—is the only potential curative treatment. PD is a complex procedure, comprising multiple components and pre-, peri-, and post-operative interventions and co-interventions, such as biliary stenting, use of somatostatin analogues, and dietary interventions, and confers significant morbidity (~70% with 3% mortality) [2,3]. Long-term survival after PD has not significantly improved despite advancements in chemotherapy [4,5,6,7], peri-operative care [8,9,10], and advanced surgical techniques such as vascular resection and reconstruction [11,12,13,14,15]. Therefore, there is an urgent need for further high-quality research to improve patient outcomes in this clinical area.

Although randomised controlled trials (RCTs) represent the optimal method for assessing the effectiveness of interventions [16,17], they are not straightforward to design or conduct in a surgical setting. Surgery is a complex intervention, with multiple components and co-interventions that can be delivered in different ways by different surgeons. For example, there are several acceptable ways in which PD can be delivered [18,19,20]. This variability in delivery of surgical procedures–and a lack of information about how they were intended to be/actually delivered in RCTs-may introduce performance bias, impact trial results, and create difficulties in replicating ‘successful’ interventions in clinical practice. One way of considering how surgical interventions should be delivered in RCTs is to apply quality assurance processes, as recommended in CONSORT-NPT (CONSORT statement for non-pharmacological treatments) guidance. Quality assurance is an umbrella term encompassing (i) description(s) of the intervention and its components, (ii) consideration of whether or not intervention delivery should be standardised, (iii) monitoring whether the standards were met (i.e., adherence), and (iv) consideration for the expertise participating surgeons and centres. The concept of quality assurance is particularly pertinent in RCTs involving complex procedures such as PD.

This narrative review aimed to explore and summarise reporting of quality assurance measures for surgical interventions in RCTs, using PD as an exploratory case study. A structured narrative approach was chosen because the focus of this review is the reporting and conceptualisation of operative quality assurance rather than intervention effects. Quality assurance measures in surgical RCTs are heterogeneous, largely qualitative, and inconsistently defined, which limits the feasibility of meaningful quantitative synthesis or meta-analysis. A structured narrative approach was therefore considered methodologically appropriate to map and interpret reporting practices across contemporary trials involving pancreatoduodenectomy. 

## 2. Materials and Methods

This narrative review aimed to summarise how contemporary randomised controlled trials (RCTs) involving pancreatoduodenectomy (PD) describe operative technique, standardisation, and quality-assurance processes. Although not conducted as a formal systematic review, the approach drew on structured search and selection principles to identify contemporary RCTs relevant to current PD trial practice.

### 2.1. Selection Criteria

English-language studies published between 1 January 2020 and 10 July 2024, involving adult patients undergoing PD, were included with the intention of capturing contemporary trial practice, reflecting current surgical techniques, peri-operative care pathways, and reporting expectations in modern pancreatic surgery trials. Earlier trials were important in the historical development of surgical quality assurance but frequently predate contemporary guidance on trial reporting and operative transparency, including recommendations outlined in CONSORT-NPT. As the aim of this review was to explore how quality assurance processes are reported in current RCT practice, restricting the search to more recent trials was considered appropriate. Studies that were non-randomised, pre-clinical, cadaveric, non-English, or published only as abstracts were not included due to insufficient methodological detail. Trials involving other pancreatic procedures (e.g., distal or total pancreatectomy) were excluded.

### 2.2. Search Strategies

Exploratory searches of Medline, the Cochrane Central Register of Controlled Trials, and the Cochrane Database of Systematic Reviews were used to identify contemporary RCTs. Searches were developed around the terms ‘pancreatoduodenectomy’ and ‘randomised control trial’ and combined using the Boolean ‘AND’ operators. Reference lists of included studies, related article functions in PubMed, and the International Study Group of Pancreatic Surgery living evidence map were also consulted to locate additional relevant trials.

### 2.3. Study Selection

Titles and abstracts were reviewed for relevance to PD and RCT methodology. Full texts were retrieved when the abstract did not provide sufficient information or when inclusion was uncertain. Selection was guided by relevance to operative reporting, trial design, and methodological considerations rather than rigid systematic criteria. The literature identification and selection process are illustrated in Supplementary Figure S1.

### 2.4. Data Extraction

A structured template was used to guide consistent extraction of key methodological characteristics from trial publications, associated protocols, and Supplementary Materials where available. Extracted information focused on study characteristics and aspects of quality assurance relevant to surgical trial conduct. Findings were synthesised narratively with the aim of identifying overarching themes and variations in reporting rather than quantifying effect sizes or producing pooled estimates.

### 2.5. Extracted Study Characteristics

Study characteristics were recorded, including the number of centres and patients, the country of study, and the nature of the intervention and comparator under investigation.

### 2.6. Quality Assurance

Information about quality assurance was collected in the following categories, as recommended by CONSORT-NPT.

#### 2.6.1. Framework for PD Component Description 

Using a published framework [21] PD was conceptualised as comprising 40 component steps spanning access, dissection, resection, haemostasis, reconstruction, and closure. This framework was developed by four consultant pancreatic surgeons (SP, AS, SS, JAGM). Trial reports and Supplementary Materials were reviewed to identify which components, if any, were explicitly described.

#### 2.6.2. Standardisation of Component Parts

The review examined whether trials described attempts to standardise aspects of PD. Standardisation was interpreted as any effort to create uniformity across participating surgeons, such as operative manuals, mandatory or prohibited techniques, or prespecified procedural requirements. Explicit terminology (e.g., “standardised,” “uniform,” “mandatory,” “prohibited”) and descriptions of standardisation strategies were noted. Where trials stated that surgeons could operate according to their institutional “standard practice,” this was interpreted as allowing procedural flexibility.

#### 2.6.3. Assessment of Adherence to Operative Standards

For each included RCT, we examined whether any method was used to monitor how PD was delivered in practice. This included review of operative notes, intra-operative photographs, intra-operative videos, or any other mechanism described by trial authors. The intention was to capture how trials attempted to ensure that reported standards, where described, were followed during surgery.

#### 2.6.4. Assessment of Centre and Surgeons Expertise

Details regarding the surgeon and centre experience were reviewed descriptively. This included whether trials reported operative volumes, minimum participation criteria, credentialing processes, or other markers of expertise. The goal was to understand how contemporary PD trials approach the issue of surgical proficiency and whether expertise was considered as part of trial design.

### 2.7. Data Analysis

Study characteristics and quality-assurance practices were summarised descriptively to identify recurring patterns, areas of variation, and gaps in reporting across contemporary PD RCTs. Proportions or counts were presented only to help illustrate broad trends; no quantitative pooling or meta-analysis was undertaken. The emphasis of the analysis was to explore how operative technique, standardisation, adherence, and surgeon/centre expertise are currently considered and reported in PD trials, rather than to compare outcomes across studies.

## 3. Results

### 3.1. Study Characteristics

A total of 158 articles were identified through database searches, with an additional 31 identified via the ISGPS evidence map [22], of which 37 RCTs were included in the final analysis. All studies were parallel two-arm RCTs reporting outcomes in 5659 patients across 16 countries (Table 1 and Supplementary Table S1). Twenty studies randomised fewer than 100 patients, and 27 were single centres. Most (*n* = 22) evaluated intra-operative interventions, of which 18 related to a technical aspect of PD (e.g., Blumgart pancreatic anastomosis versus Cattell-Warren pancreatic anastomosis) [23].

#### Description of PD

None of the included RCTs described all 40 component parts, and four did not describe any. The median number of components described was 4 (interquartile range 2–8), with one RCT describing 24 (Supplementary Table S2). Commonly reported components included the method of pancreatic reconstruction (*n* = 24), insertion/use of abdominal drains (*n* = 19), method of hepaticojejunostomy (*n* = 15) and gastrojejunostomy (*n* = 15), and insertion of a pancreatic stent (*n* = 12).

There was variation in how components were described. Supplementary Table S3 provides verbatim descriptions of selected PD components, using three RCTs comparing methods of pancreatic anastomosis as an example of the heterogeneity.

### 3.2. Standardisation of the Intervention and Component Parts

Nine trials mentioned some form of standardisation of PD [24,25,26,27,28,29,30,31,32,33]. Of these, one provided methods of standardisation, using an operative manual that was distributed to participating surgeons [23,34].

Across the included trials, attempts to standardise operative delivery most commonly focused on the specific procedural component under investigation, such as pancreatic anastomosis technique, reconstruction route, or abdominal drain strategy. In contrast, many other operative steps involved in pancreatoduodenectomy were either variably described or left to the discretion of the operating surgeon, indicating that trial protocols typically defined or constrained only selected procedural elements rather than the entire procedure.

Reporting of flexibility in intervention delivery varied between trials. For example, one RCT allowed flexibility in 24 of the 26 components described, whereas four trials did not permit any flexibility. For example, Toyama et al. permitted flexibility in the placement of intra-abdominal drains, stating, “The number, types, and locations of intra-abdominal drainage tubes were determined according to operating surgeons’ preferences” [35]. This contrasts with Busquets et al., where the number and location of drains were specified: “In both groups, two drains were placed close to the pancreaticojejunal anastomosis (*n* = 2) and one posteriorly to the hepaticojejunal anastomosis (Bellovac, Wellspect, HealthCare, Möndal, Sweden)” [36].

Twelve out of 24 trials describing the formation of the pancreatic anastomosis did not allow any flexibility in this component; the depth of description varied. For example, Seradilla-Martín et al. provided a precise description of the pancreatic anastomosis, stating “The PJ was performed in one layer using a continuous barbed suture (V-LokTM 3/0; Medtronic, Dublin, Ireland) in the posterior and anterior sides of the anastomosis or using a two-layer, duct-to-mucosa, end-to-side technique, with interrupted sutures using monofilament sutures, according to the individual surgeon’s preference” [31]. Other trials were less descriptive of the pancreatic anastomosis, such as the trial by Busquets et al., stating “Reconstruction was performed on a single loop, starting with pancreatic anastomosis. Pancreatic, biliary, and gastric anastomoses were performed in a retrocolic position. Duct-to-mucosa pancreatojejunostomy was the first choice for all patients” [36].

### 3.3. Adherence to Operative Standards

Only three trials described any monitoring of adherence to the reported description/standardisation of PD. All three used operative photographs, although further details of how assessments were undertaken were not provided [23,24,37].

#### Expertise of Centres and Surgeons

Six trials specified a minimum centre volume of PD, with two specifying ≥ 40 PD per year [38,39], two ≥ 50 PD per year [24,31], and two ≥ 100 PD per year [40,41].

Two trials specified minimum surgeon volumes of at least 50 [39] and 60 [42] major pancreatic resections per year. Another specified that participating surgeons needed to be certified by the Japanese Society of Gastroenterological Surgery and had at least 15 years’ experience [43]. In two studies, formal assessment of surgeons was undertaken through review of operative photographs, prior to participation [37,44]. Photographs were centrally assessed by trial leads. One RCT also required surgeons to submit unedited PD videos for central assessment prior to participation, to ensure they were ‘adeptly done by independent expert evaluation’ [24].

## 4. Discussion

This narrative review summarises reporting of quality assurance processes in PD within published RCTs, using CONSORT-NPT recommendations as reporting standards. The majority of included studies were single-centre and recruited fewer than 100 patients. Component parts of a PD were described poorly. Four RCTs did not describe how any component parts of a PD were undertaken. Nine trials standardised PD delivery in some way, and methods to monitor adherence were used in only three studies. Surgeon and centre expertise were also infrequently reported. These limitations introduce uncertainty into how PD was actually delivered within the included RCTs, limiting the accurate interpretation of results and their subsequent implementation into clinical practice. Supplementation of CONSORT-NPT with recommendations specific to surgery may help to resolve these issues and improve the application of quality assurance processes in this context.

A recent study found that both the quantity and quality of RCTs in pancreatic surgery have increased over the past 30 years [45]. However, it did not specifically investigate the issue of performance bias in surgical RCTs or the potential solution of applying quality assurance measures to surgical interventions. This is particularly relevant given that applying CONSORT-NPT recommendations can be challenging, as has already been found in other reviews [46,47]. For example, CONSORT-NPT suggests that ‘precise’ details of the intervention and comparator should be provided, but the meaning of this term is not explained. Another limitation of CONSORT-NPT is that it focuses solely on the intervention and comparator. In surgical RCTs, the intervention and comparator might be components of a procedure (rather than an entire procedure) or a co-intervention, yet it is nevertheless important to consider quality assurance of other procedural components and co-interventions, which also have the potential to influence outcomes.

Future iterations of CONSORT-NPT should consider these issues, or specific guidance for invasive procedures such as surgery may be warranted. The limited reporting of surgical quality assurance measures may reflect several practical barriers to their implementation within RCTS of complex procedures such as pancreaticoduodenectomy. Processes such as the development of operative manuals, surgeon credentialing, and monitoring of adherence through intra-operative photography or video review require additional time, infrastructure, and logistical coordination, which may limit their routine application, particularly in multicentre trials. Furthermore, the degree of operative standardisation may depend on the nature of the intervention under investigation, with trials evaluating specific technical aspects of the procedure more likely to incorporate procedural standardisation than studies investigating peri-operative or pharmacological interventions.

In addition to these practical challenges, there are conceptual difficulties in defining and applying the term ‘standardisation’ in surgical trials. One contributing factor is the inherent clinical heterogeneity of patients undergoing pancreatoduodenectomy. Heterogeneity in the underlying pathology of lesions treated within pancreatoduodenectomy trials may also influence operative decision-making and postoperative outcomes. For example, pancreatic ductal adenocarcinoma is frequently associated with a fibrotic pancreatic gland and a dilated pancreatic duct, whereas other periampullary pathologies may present with a softer gland and a smaller duct calibre. These factors are well-recognised determinants of reconstruction strategy and risk of post-operative pancreatic fistula, and therefore represent an additional source of variability across surgical RCTs.

In many pancreaticoduodenectomy RCTS, the term “standardisation” refers not to uniform delivery of the entire procedure but rather to the protocol-defined delivery of the specific procedural components related to the trial intervention. Other elements of the procedure are often permitted to vary according to the surgeon’s judgement or institutional practice. In this context, different forms of standardisation can be recognised in surgical trials. These include protocol-defined procedural delivery, where operative manuals or predefined protocols specify how key components of a procedure should be undertaken; component-level standardisation, where only the operative step under investigation is specified within the trial protocol; and implicit standardisation, achieved through surgeon or centre expertise without formally mandating specific technical steps.

It is difficult to imagine how it is possible (or necessary) to standardise all components of complex interventions such as pancreatoduodenectomy, especially when delivered in a pragmatic setting by multiple centres and surgeons. Although strict standardisation may be possible in a single centre trial investigating efficacy, maintaining consistency in the delivery of all intervention and co-intervention components across larger multicentre studies is unlikely to be feasible and may compromise generalisability. A further challenge is the monitoring of adherence to intervention delivery, which has been done successfully in several multicentre trials. For example, PANASTA [23], which compared methods of pancreatic reconstruction following PD, used an operative manual to standardise both intervention and comparator, and intra-operative photos were reviewed to assess adherence during the internal pilot phase. Although these methods have been replicated in other RCTs (e.g., the ROMIO study), they are labour and resource-intensive, which may explain the limited application of intervention adherence assessments in surgical trials.

This review has several limitations. First, some trials may have considered intervention description, standardisation, and adherence, but did not report these details in their publications. Word limits may have led to omission of key quality-assurance information, and although published protocols or Supplementary Materials, including Supplementary Table S1 [48,49,50,51,52,53,54,55,56,57,58,59,60], should mitigate this, these sources also lacked sufficient detail when reviewed.

Second, the review assumed that standardisation had not occurred unless explicit terminology (e.g., “standardise,” “uniform”) or clear methods were described. The deconstruction of PD into component steps was undertaken by four surgeons and was not validated more broadly. Importantly, the purpose of deconstructing PD into component steps is to facilitate transparent reporting of which components are mandated, flexible, or monitored within individual studies, rather than to require rigid standardisation of every element within RCTs.

Third, although PD was broken down into 40 components, it is neither necessary nor practical to expect routine reporting of every element in RCTs. More rigorous reporting is likely to be required for trials evaluating technical interventions compared with pharmaceutical studies or routine clinical practice. Some components are irrelevant to particular interventions or outcomes, for example, documenting the method or extent of duodenal Kocherisation in a drug-evaluation RCT.

There is a pressing need for an international consensus on PD deconstruction and associated co-interventions. Using such a framework, future pancreatic RCTs should determine which components require reporting, which should be mandated or flexible, and whether adherence should be monitored. The degree of standardisation needed will vary with factors such as the number of participating surgeons and centres, whether the design is pragmatic or explanatory, and whether the intervention is novel or an established practice.

## 5. Conclusions

This narrative review highlights significant deficiencies in the application of quality-assurance processes to PD, a complex surgical procedure. These gaps hinder translation of trial findings into clinical practice because the operative approach is often unclear, reducing confidence in the results. A broader international agreement on core PD components and co-interventions is required. Further work should update CONSORT-NPT guidance or develop procedure-specific guidance for invasive interventions such as surgery. In addition to providing practical examples of quality-assurance application in surgical RCTs, future guidance should also describe factors that influence the level of QA required. Addressing these issues will help minimise performance bias and enhance the generalisability of surgical trials.

## Figures and Tables

**Table 1:** Summary of characteristics of included studies.

Characteristic		Number of RCTs (*n* = 37)
Year of publication	2020	16
	2021	9
	2022	6
	2023	6
Number of centres	Single centre	27
	Multicentre	11
Number of patients randomised	<50	6
	50–99	14
	100–199	8
	200–299	5
	>300	4
Phase of trial	II	2
	III	35
Type of intervention under investigation	Pre-operative intervention	2
	Intra-operative intervention	22
	Post-operative intervention	13

## Data Availability

No new data were created or analysed in this study.

## Supplementary Materials

The following supporting information can be downloaded at: https://www.mdpi.com/article/10.3390/jcm15062455/s1. Supplementary Figure S1: Literature identification and selection process; Supplementary Table S1: Description of RCTs included in narrative review, including studies cited in references [48,49,50,51,52,53,54,55,56,57,58,59,60]; Supplementary Table S2: Provision of descriptions of the component parts of PD across the included RCSTs., Supplementary Table S3: Verbatim descriptions of selected component parts in the three trials comparing the method of pancreatic reconstruction.

## Abbreviations

The following abbreviations are used in this manuscript:

CONSORT	Consolidated Standards of Reporting Trials
PD	Pancreaticoduodenectomy
RCT	Randomised Controlled Trial
SPIRIT	Standard Protocol Items: Recommendations for Interventional Trials
COREQ	Consolidated Criteria for Reporting Qualitative Research
PRISMA	Preferred Reporting Items for Systematic Reviews and Meta-Analysis
ERAS	Enhanced Recovery After Surgery
