# Supplementary material for: Reporting Standards and Quality Assurance Methods for Pancreatoduodenectomy in Randomised Controlled Trials: A Structured Narrative Review
Source: J Clin Med. 2026 Mar 23;15(6):2455. doi: 10.3390/jcm15062455 (PMC13028529; doi:10.3390/jcm15062455)
Supplement: Supplementary file 1 [file jcm-15-02455-s001.zip › jcm-4142481-supplementary.pdf]

Supplementary Figure S1. Literature Identification and Selection Process

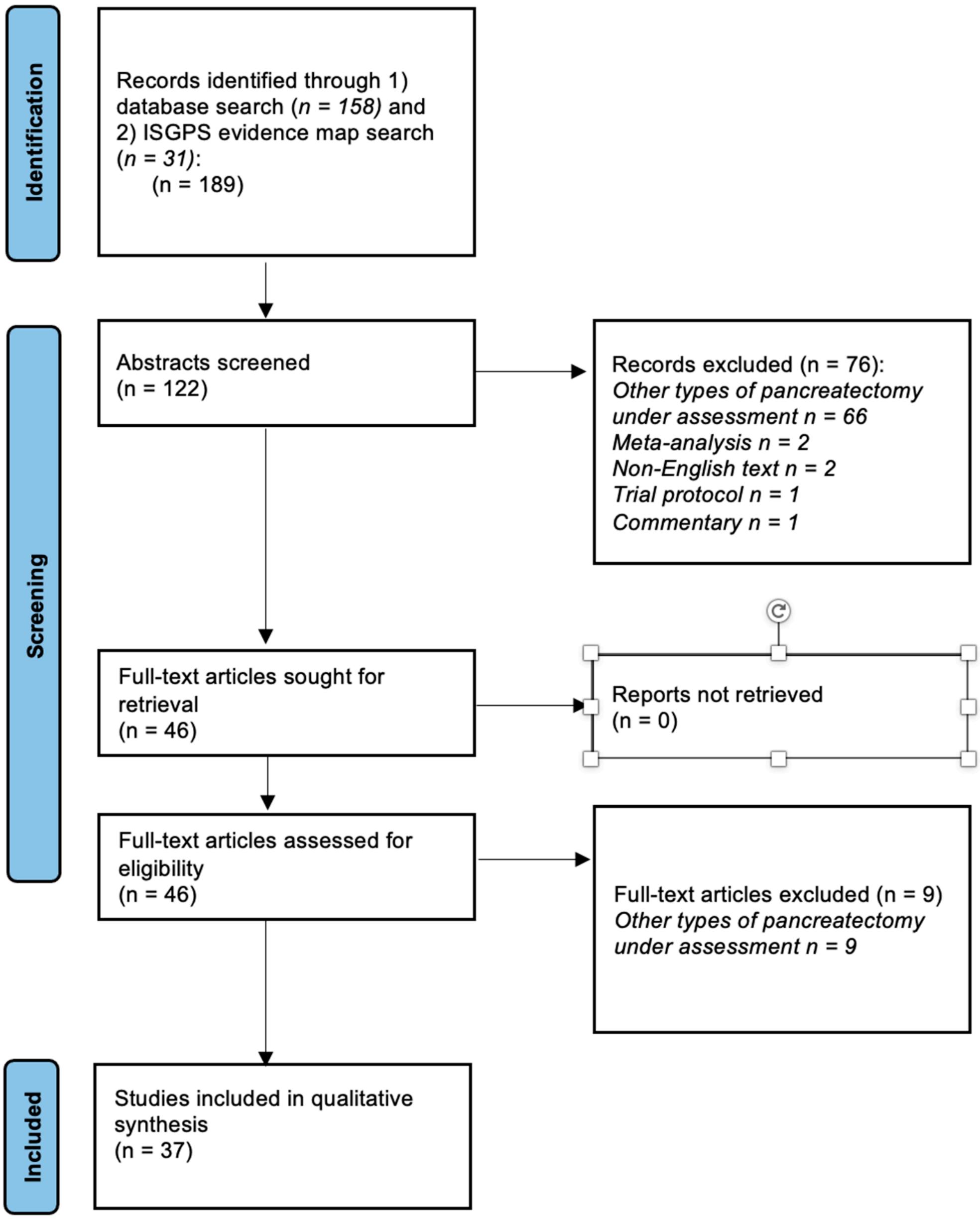

Supplementary Table S1. Description of RCTs included in narrative review

Study number	Author	Year of publication	Country	Multicentre	Phase of study	Number of patients	Intervention	Control	Primary outcome
1	Alston James[48]	2020	USA	No	II	44	Thunderbeat ™	Standard electrosurgery	Estimated blood loss
2	Andrianello[42]	2020	Italy	No	III	72	Pancreatogastrostomy	Pancreatojejunostomy	Postoperative pancreatic fistula
3	Bergeat[49]	2020	France	No	III	111	Routine nasogastric tube insertion	No nasogastric tube as routine	Occurrence of Clavien Dindo complication grade >2
4	Busquets[36]	2022	Spain	No	III	79	Classic pylorus-resecting Whipple	Pylorus-preserving pancreaticoduodenectomy	Delayed gastric emptying
5	Cai[40]	2022	China	No	II	90	Pancreatic anastomosis stent	No stent	Postoperative pancreatic fistula
6	Chen[18]	2023	China	No	III	90	Dexmedetomidine	Normal saline	Neutrophil to lymphocyte ratio
7	De Pastena[19]	2020	Italy	No	III	190	Alexis wound protector	Standard operative draping with no wound protector	Incidence of surgical site infection
8	Eguchi[43]	2020	Japan	No	III	53	Pancreatogastrostomy	Pancreatojejunostomy	Delayed gastric emptying
9	El-Haddad[20]	2021	Egypt	No	III	60	Endoscopic biliary drainage	Percutaneous biliary drainage	Operative difficulty

10	Folwarski[50]	2021	Poland	No	III	40	Probiotic	Standard oral intake without probiotic	GI function
11	Gehrig[25]	2020	Germany	No	III	86	LigaSure™ Impact	Conventional dissection methods	Operating time
12	Halloran[23]	2022	UK	Yes	III	236	Blumgart anastomosis	Cattell-Warren anastomosis	Postoperative pancreatic fistula
13	Ishii[51]	2022	Japan	Yes	III	202	Tranexamic acid	Placebo (normal saline)	Blood loss
14	Kapritsou[52]	2020	Greece	No	III	85	Enhanced recovery after surgery protocol	Standard postoperative recovery	Pain score
15	Klaiber[27]	2020	Germany	No	III	188	Pylorus-preserving pancreaticoduodenectomy	Pylorus-resecting pancreaticoduodenectomy	Delayed gastric emptying
16	Klotz[28]	2020	Germany	Yes	III	248	Intravenous patient-controlled analgesia	Epidural anaesthesia	Incidence of gastrointestinal complications
17	Kumar[53]	2023	India	No	III	44	Rectal indomethacin at induction of anaesthesia	Standard of care	Postoperative hyperamylasaemia
18	Lin[29]	2023	China	No	III	120	Prophylactic irrigation-suction of pancreatic anastomosis	Routine gravity drainage of pancreatic anastomosis	Postoperative pancreatic fistula
19	Liu[30]	2023	China	No	III	134	Parecoxib	Standard analgesia	Pain score
20	Park[54]	2020	Korea	No	III	38	Needle electrical twitch obtaining intramuscular stimulation postoperatively	Standard of care	

21	Serradilla-Martín[31]	2023	Spain	No	III	64	Hemopatch placement around pancreatic anastomosis	Pancreatic anastomosis left bare	Postoperative pancreatic fistula
22	Singh K[55]	2021	India	No	III	50	Pancreaticojejunostomy stent	No stent	Postoperative pancreatic fistula
23	Singh H[32]	2020	India	No	III	40	Clamping of common hepatic duct	No clamping	Surgical site infection incidence
24	Tangtawee[56]	2021	Thailand	No	III	68	Omental roll up on pancreatic anastomosis	No omental roll up	Postoperative pancreatic fistula
25	Toyama[35]	2020	Japan	Yes	III	214	Retrocolic reconstruction	Antecolic reconstruction	Delayed gastric emptying
26	Tumas[33]	2020	Lithuania	No	III	70	Nutritional intervention with immunonutrition	No intervention	C-reactive protein
27	Van Roesse[38]	2021	Netherlands	Yes	III	128	Bivalving specimen	Axial slicing	Level of certainty of site of origin
28	Wang M[24]	2021	China	Yes	III	656	Laparoscopic pancreatoduodenectomy	Open pancreatoduodenectomy	Postoperative length of stay
29	Wang Z[44]	2021	China	No	III	153	Extended lymphadenectomy	Standard lymphadenectomy	2-year overall survival
30	Welsch[37]	2022	Germany	Yes	III	407	Falciform ligament wrap	No wrap	Clinically relevant postpancreatectomy haemorrhage
31	Yasukawa[39]	2020	Japan	No	III	80	High dose enzymes	Low dose enzymes	Incidence of non-alcoholic fatty liver disease
32	Cao[41]	2021	China	Yes	III	205	Postoperative somatostatin infusion	Routine postoperative care	Clinically-relevant postoperative pancreatic fistula
33	D'Angelica[57]	2023	USA	Yes	III	778	Pipercillin-Tazobactam	Cefoxitin	Postoperative surgical site infection
34	Di Mola[58]	2020	Italy	No	III	48	Large jejunal incision for	Small jejunal incision for	Postoperative pancreatic fistula

							pancreatic anastomosis	pancreatic anastomosis	
35	Liu[59]	20 21	China	No	III	120	Early oral feeding	Early nasojejunal nutrition	Delayed gastric emptying
36	Yamaguc hi[26]	20 20	Japan	No	III	56	Rikkunshito	No intervention	Delayed gastric emptying
37	Dai [60]	20 22	China	Yes	III	312	Early drain removal	Late drain removal	Incidence of Clavien-Dindo grade 2-4 complications

Supplementary Table S2. Provision of descriptions of the component parts of PD across the included RCSTs.

Component		Paper number																																				
		1	2	3	4	5	6	7	8	9	10	11	12	13	14	15	16	17	18	19	20	21	22	23	24	25	26	27	28	29	30	31	32	33	34	35	36	37
Preoperative considerations	Preoperative biliary stenting																																					
	Review of most up to date cross-sectional imaging																																					
Before skin incision	Positioning																																					
	Prophylactic antibiotics																																					
Incision																																						
Dissection/assessment	Kocher manoeuvre																																					
	Mobilisation of right colon																																					
	Entry into the lesser sac																																					
	Trunk of Henle																																					

	division, and assessment of infra-pancreatic SMV and uncinate process																																					
	Cholecystectomy																																					
	Dissection of hepatoduodenal ligament																																					
	Dissection of GDA																																					
	Assessment of hepatic artery flow ('Pinch test')																																					
	Decision to proceed with resection																																					
**Resection**	Division and transf																																					

ixion of gastroduodenal artery (+/- right gastric artery)																																					
Division of common bile duct																																					
Dissection of duodenum from ligament of Treitz																																					
Division of stomach																																					
Division of proximal jejunum																																					
Division of pancreatic neck																																					
Resection approach (e.g. artery first, uncin																																					

	ate first)																																					
	Dissection of uncinate process from retropancreatic tissue																																					
	Removal of specimen																																					
	Lymph node sampling																																					
**Haemostasis**																																						
**Reconstruction**	Pancreatico-jejunostomy/gastrostomy																																					
	Use of pancreatic stent																																					
	Hepaticojejunostomy																																					
	Gastrojejunostomy																																					
	Vascular recon																																					

	struction																																					
	Washout																																					
**Insertion of surgical adjuncts**	Insertion of abdominal drain(s)																																					
**Closure**	Fascial closure																																					
	Skin closure																																					
**After closure**	Application of dressings																																					
	Postoperative antibiotics																																					
	Postoperative venous thromboembolism prophylaxis																																					
	Use of somatostatin analogues																																					

Postoperative pain control plan																																					
Postoperative feeding plan																																					

Supplementary Table S3. Verbatim descriptions of selected component parts in the three trials comparing method of pancreatic reconstruction

	Study ID		
Component	**2**	**8**	**12**
Pancreatico-jejunostomy/gastrostomy	'*Pancreatojejunostomy was performed according to the Cattel-Warren duct-to-mucosa technique using polyester-interrupted sutures (3/0 or 4/0) for the outer layer and polypropylene-interrupted sutures (5/0 or 6/0) for the inner layer. Pancreaticogastrostomy was performed according to the Bassi technique*'	'[Pancreaticogastrostomy] *was performed according to the method described by Ohigashi et al…*'<br><br>'[Pancreaticojejunostomy] *was performed according to the method described by Kakita et al., with some modifications…*'	'*Reconstruction of the pancreatic remnant following pancreato-duodenectomy (either Kausch-Whipple or pylorus-preserving) using a "Blumgart" method of pancreatico-jejunostomy*'<br><br>'*Reconstruction of the pancreatic remnant following pancreato-duodenectomy (either Kausch-Whipple or pylorus-preserving) using a "Cattell-Warren" method of pancreatico-jejunostomy*'
Use of pancreatic stent	'*Regardless of the randomization group, a transanastomotic stent consisting of a 5-, 6-, or 7.5-Fr PankreaPlus polyvinyl catheter (Peter Pflugbeil Gmbh Medizinische Instrumente) was placed. The stent was externalized through the pancreatobiliary limb beyond the hepaticojejunostomy employing the Wietzel tunnel technique. The largest stent that could traverse the anastomosis without generating tension was used*'	'*a polyethylene pancreatic tube was introduced into the main pancreatic duct and fixed to the main pancreatic duct using a 5–0 absorbable suture*'	'*The use of a pancreatic duct stent is mandatory in all patients*'
Hepaticojejunostomy	'*hepaticojejunostomy… will be carried out as usual*'	*Not described*	*Not described*
Gastrojejunostomy	'*duodenojejunostomy (in case of Longmire-Traverso PD) or gastrojejunostomy (in case of Kausch-Whipple PD), will be carried out as usual*'	*Not described*	*Not described*
Insertion of abdominal drains	'*Two easy-flow drains were placed in the proximity of the pancreatic and biliary anastomoses*'	'*The amylase in drains was measured on days 1–3 after surgery and repeated later if levels continued to be higher than the upper limit of the plasma level*'<br><br>The location, size and number of drains was not specified, nor any flexibility permitted/prohibited.	'*Surgical drains (of any description) must be employed and should be left in place for a minimum of 3 days after surgery*'
Use of somatostatin analogues	'*No prophylactic octreotide was used*'	*Not described*	'*All patients will receive an initial dose of 100 μg of octreotide before surgery. Octreotide should then be administered at 100 μg three times a day*

	subcutaneously on post-operative days 1 to 7'
